# Bruton’s Tyrosine Kinase and Its Isoforms in Cancer

**DOI:** 10.3389/fcell.2021.668996

**Published:** 2021-07-08

**Authors:** Xianhui Wang, Leila Kokabee, Mostafa Kokabee, Douglas S. Conklin

**Affiliations:** Department of Biomedical Sciences, Cancer Research Center, State University of New York, Rensselaer, NY, United States

**Keywords:** cancer, BTK, PI3K, kinase inhibitor, metastasis

## Abstract

Bruton’s tyrosine kinase (BTK) is a soluble tyrosine kinase with central roles in the development, maturation, and signaling of B cells. BTK has been found to regulate cell proliferation, survival, and migration in various B-cell malignancies. Targeting BTK with recently developed BTK inhibitors has been approved by the Food and Drug Administration (FDA) for the treatment of several hematological malignancies and has transformed the treatment of several B-cell malignancies. The roles that BTK plays in B cells have been appreciated for some time. Recent studies have established that BTK is expressed and plays pro-tumorigenic roles in several epithelial cancers. In this review, we focus on novel isoforms of the BTK protein expressed in epithelial cancers. We review recent work on the expression, function, and signaling of these isoforms and their value as potential therapeutic targets in epithelial tumors.

## Introduction

Bruton’s tyrosine kinase (BTK) is a member of the TEC family, a group of cytoplasmic, non-receptor tyrosine kinases including BTK, ITK, BMX, and TEC (Smith et al., 2001). The kinase was initially identified in the 1990s as a novel non-receptor protein tyrosine kinase mutation that is responsible for human X-linked agammaglobulinemia (XLA) disease (Tsukada et al., 1993; Vetrie et al., 1993). The *BTK* gene is located on the X chromosome (Xq21.33–Xq22). Mutations in BTK lead to defective maturation and development of B cells, which is the root cause of the XLA disorder. Significantly diminished numbers of mature B cells lead to the XLA immunodeficiency syndrome first described by Dr. Ogden C. Bruton at Walter Reed Army Hospital in 1952 (Bruton, 1952). The condition is manifested in predominantly young male patients who have frequent severe infections and exhibit nearly complete loss of B cells and dramatically decreased levels of immunoglobulins in the bloodstream (Hendriks et al., 2011).

Bruton’s tyrosine kinase is predominantly expressed in hematopoietic cells including erythroid progenitors and myeloid cells (Mohamed et al., 2009). Accordingly, no evidence has been found to suggest that the cause of XLA occurs anywhere but within the B-cell lineage in these patients. Studies have established that BTK has a crucial role for B-cell development, differentiation, survival, and signal transduction (Aoki et al., 1994; Kawakami et al., 1994; Khan et al., 1995). In mice, a single point mutation in BTK causes a less severe X-linked immunodeficiency (XID) phenotype, and the effects of this mutation appear to be limited to the B-cell population (Rawlings et al., 1993; Thomas et al., 1993). Recently, results from a number of studies point to BTK’s signaling role as critical to the survival of B-cell leukemia and lymphoma cells (Buggy and Elias, 2012; Hendriks et al., 2014). These studies provide the rationale for targeting BTK in the treatment of B-cell malignancies.

## Isoforms of Bruton’s Tyrosine Kinase in Epithelial Tumors

**FIGURE 1:**
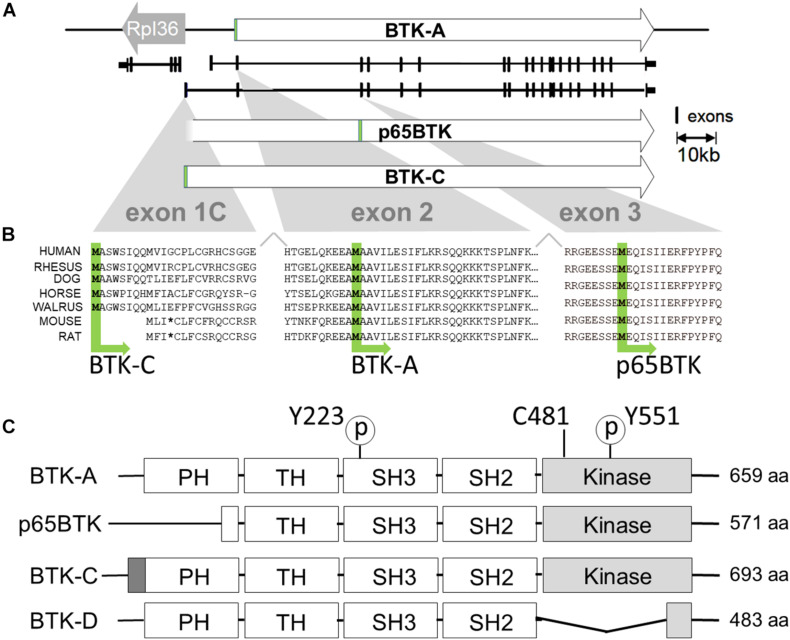
Transcription initiation and translation initiation sites of BTK isoforms. **(A)** X chromosome map of the BTK locus. BTK-A is the isoform expressed in B-cells. BTK-C exon1 transcription initiates 255 bp from the start site of the ribosomal protein *L36a* gene and is the predominant form expressed in some epithelial tumor cell lines. **(B)** Exon1C encodes a phylogenetically conserved 34 amino acid extension at the N terminus. Translational initiation of p65BTK occurs at a conserved methionine codon in exon 3. Some sequence has been omitted from exon2 for clarity. **(C)** Structures of BTK isoforms with motifs shown. Activating phosphorylation sites pY223 and pY551 are shown. Cysteine at 481 is the binding site for BTK inhibitors ibrutinib, AVL-292 (spebrutinib) and acalabrutinib. p65BTK has a partial PH domain. BTK-C has a 34-amino acid extension of PH domain. BTK-D has a partially deleted kinase domain.

Computation-based gene prediction and RNA-seq approaches have identified additional isoforms of BTK in humans and other mammals (Figure 1). Surprisingly, these isoforms appear to be predominantly expressed in tissues and epithelial tumors unrelated to B-cell and B lymphocyte malignancies. Epithelial tissues give rise to approximately 80 to 90% of all tumors and are in turn responsible for commensurate percentage of cancer deaths worldwide [NCI-SEER, 2014; Surveillance Research Program [SRP], 2021]. These tumors are derived from tissues of the breast, prostate, and lung, among others, and are often driven by aberrant increased signaling relevant in the original epithelial tissue (Hanahan and Weinberg, 2011). Given the role that BTK plays in survival of B-cell lymphomas, the expression of other BTK kinases in this large cancer class may represent an important therapeutic vulnerability.

Bruton’s tyrosine kinase isoforms expressed in epithelial tumors differ from the original isoform of BTK in B cells (which we refer to BTK-A for clarity, ENST00000308731.7), which was cloned in 1993, is 659 amino acids long, and has a molecular weight of 77 kDa. Its structure includes an N-terminal pleckstrin homology (PH) domain followed by a Tec homology (TH) domain, a Src-homology 3 (SH3) domain, an SH2 domain, and a C-terminal kinase domain. BTK-A is the most widely studied isoform of BTK and is expressed highly in the hematopoietic lineage. In 2016, the Grassilli group reported the identification and characterization of p65BTK, a truncated isoform that is expressed in colon carcinoma cell lines and tumors. p65BTK is composed of 571 amino acids with an apparent molecular weight of 65 kDa. Compared with BTK-A, p65BTK lacks most of the N-terminal PH domain. Its open reading frame starting codon is in exon 3 (ATG) starting at a conserved methionine at position 89 of the BTK-A sequence (Grassilli et al., 2016). In 2013, Eifert et al. identified a novel isoform of BTK (BTK-C) in breast cancer. The name was derived from the fact that the 5′ end of the transcript has an alternative first exon identical to a computationally predicted (GNOMON) sequence named BTK-cra-C. The BTK-C isoform was initially identified in a tyrosine kinome-wide RNAi screen for survival factors in breast cancer cells. Subsequent studies indicated that BTK-C is frequently expressed in both cell lines and epithelial tumors. BTK-C is the predominant isoform expressed in breast tumor cells and is transcribed from an alternative promoter. The encoded protein possesses a 34 amino acid amino-terminal extension compared with BTK (BTK-C, ENST00000621635.4) (Eifert et al., 2013). BTK-C is also expressed in prostate cancer cells (Kokabee et al., 2015). In 2005, the Feldhahn group identified a BTK splice variant isoform that acts as a dominant-negative allele. The expression of this variant is capable of suppressing pre-B-cell receptor signaling and BTK-dependent differentiation of leukemia cells. This isoform lacks the kinase domain and has 483 amino acids with a total molecular weight of 52 kDa (BTK-D, ENST00000372880.5) (Feldhahn et al., 2005).

## Genetics and Structure of Bruton’s Tyrosine Kinase Isoform C

As shown in Figure 1, BTK-C is a phylogenetically conserved isoform of the kinase. The *BTK-C* gene spans approximately 52.5 kb in the Xq22-23 region. The first exon of BTK-C (exon 1C) contains 72 nucleotides and initiates approximately 15 kb upstream from the first exon of BTK-A (Figure 1A). BTK-C transcription initiates at a site 255 bp from the transcriptional initiation site of the ribosomal protein *L36a* gene. The transcription of the two genes is bidirectional. A putative transcription start site sequence for BTK-C is found 200 bp upstream of the start of exon 1C in a CpG island. Several predicted transcription factor binding sites exist in this region. They include binding sites for the transcription factors ETS, AP2, AHR, and HOXA7. Since BTK-A and BTK-C have different first exons, they use different donor sites to splice to a common acceptor site in exon 2 (Eifert et al., 2013).

Transcriptional initiation at exon 1C start site likely encodes different protein isoforms. Immunoblots of tissues indicate that both the BTK-C 80-kDa isoform and 77-kDa BTK-A isoform can be produced from this transcript. In addition, Grassilli et al. have shown that the p65BTK isoform is derived from a transcript with an alternate first exon, exon 1B, which apparently initiates very near the start of exon 1C. In the case of p65BTK, the activity of the heterogeneous nuclear ribonucleoprotein K (hnRNPK)-dependent protein controls translational initiation through an internal ribosome entry site (IRES) and start codon in exon 3 (Grassilli et al., 2016).

The BTK-C protein isoform results from initiation at the first ATG in exon 1C and is composed of 693 amino acids with a molecular weight of approximately 80 kDa, which is accurately reflected on immunoblots. The domain structure of BTK-C is similar to that of BTK-A containing in order from the N-terminus: PH domain, TH, SRC homology domains (SH domains) SH2 and SH3, and a kinase domain (Bradshaw, 2010). The only difference is the extra amino-terminal 34 amino acid extension to the BTK-A PH domain (Hyvonen and Saraste, 1997). Exon 1C sequences from other vertebrates in the UCSC Genome Browser (Kent et al., 2002) indicate that this exon is conserved (Figure 1B). The 20 organisms in both primate and vertebrate list of the genome collection all display sequence conservation within this region (Schwartz et al., 2003; Siepel et al., 2005). This includes encoded sequences in exon 1C as well as in exon 2 upstream of the start codon of the BTK-A isoform. In each organism, transcription initiation occurs divergently within a few hundred bases of the RPL 36 ribosomal protein gene. Exon 1C sequences have a range of additional amino terminal extensions between 32 and 34 amino acids. Of note, rodent genome sequences contain conserved coding sequence in exon 1C; however, a stop codon is found at the corresponding fourth position. As a result, transcripts that map to this area are not annotated in mouse genome sequences. Accordingly, the BTK-C isoform is not predicted to be expressed in rodents.

## Expression of Bruton’s Tyrosine Kinase Isoform C

**FIGURE 2:**
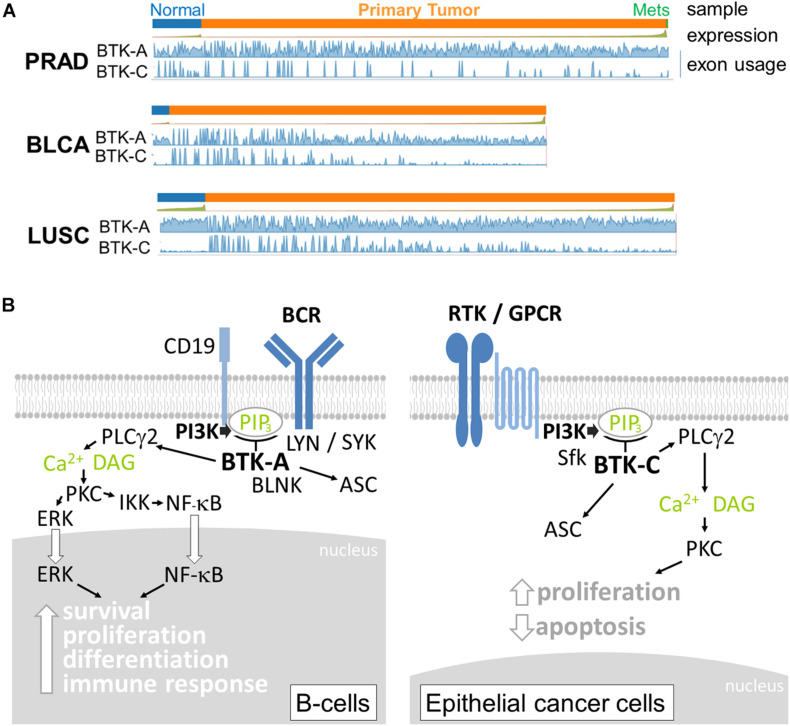
**(A)** Relative expression of Bruton’s tyrosine kinase (BTK)-A and BTK-C transcripts in epithelial tumors and normal tissue. Exon abundance for reads in TCGA Firehouse datasets for prostate adenocarcinoma (PRAD), bladder adenocarcinoma (BLCA), and lung squamous (LUSC) tumors plotted using the TSVdb web application. Each tumor type shows expression of the BTK-C transcript. Significant BTK-A transcription is also observed. **(B)** BTK isoform signaling. The BTK-A isoform is activated by B-cell receptor signaling involving the LYN and SYK kinases. PI3K activation increases PIP_3_ levels, which in turn activate BTK-A to phosphorylate PLCγ2 leading to the nuclear localization and activation of maturation transcriptional programs. In epithelial cancer cells, the key activation of PI3K can occur through diverse receptor protein tyrosine kinases, G-protein-coupled receptors, and other types of receptors, making BTK-C an important signaling node in these cells. Activation also involves SRC family kinases (Sfk). The downstream effectors of BTK-C are not completely understood at present, although the protein was isolated as a critical determinant of epithelial cancer cell survival. p65BTK (not shown) does not contain a complete PH domain and may be activated by other mechanisms.

Although expression in T cells is relatively low, BTK-A is abundantly expressed in cells derived from the hematopoietic lineage, including bone marrow cells, lymphoid, myeloid, and erythroid progenitor cells, mature B lymphocytes, mast cells, monocytes, and macrophages (Conley et al., 1994; Himmelmann et al., 1996; Niiro and Clark, 2002). BTK-A, when overexpressed in chronic myeloid leukemia (CML) and acute lymphoblastic leukemia (ALL), has been linked to imatinib resistance (Hofmann et al., 2002; Villuendas et al., 2006). Owing to their relatively recent discovery, considerably less is known about the expression of the other isoforms of BTK in epithelial tumors. Currently, information on transcript-specific expression in cancers is available online. Analysis of the expression of the BTK splice isoforms in The Cancer Genome Atlas (TCGA) database using the Tumor Splice Variant Data Base (TSV DB) indicates that virtually all epithelial tumor samples contain BTK transcripts (Sun et al., 2018). For example, the expression of the BTK-C-encoding transcript is observed in approximately 15% of normal and tumor cells in prostate, bladder, and lung squamous tumor samples (Figure 2A). BTK-A is widely expressed in the samples, although due to its high expression in B cells, it is difficult to assess whether this may be due to the presence of B-cell infiltrates.

The function of BTK isoform expression is not completely known at present. Some molecular details of BTK signaling are conserved in epithelial tumor cells. These include activation by SRC family members and downstream PLCγ2 phosphorylation (Eifert et al., 2013; Wang et al., 2016). In breast cells, BTK-C activation is inhibited by Src inhibitors dasatinib and saracatinib but not the Lyn inhibitor bafetinib (Wang et al., 2016). There are significant differences, however, between B cells and cells of epithelium origin with respect to nucleus-to-cytoplasm ratio (Fletcher, 2013) and BCR signaling and auxiliary protein expression (Li et al., 2018; Tanaka and Baba, 2020), which are likely to affect BTK signaling (Figure 2B). Similarly, there are significant differences in signaling inputs and downstream effects between the different epithelial cancers in which BTK-C is expressed. The most likely explanation for BTK isoform function in these diverse cell types stems from their critical position in PI3K signaling pathways. PI3K is among the most commonly affected pathways in epithelial tumors (Yuan and Cantley, 2008; Hopkins et al., 2018, 2020). BTK isoforms expressed in diverse cancer types are critical signaling effectors that respond to the increased production of PIP_3_ that is common to these cells. Since PI3K is activated by a wide variety of receptor types in epithelial cancers, BTK isoforms represent a common potential therapeutic target for several tumor types. For each cell type, the cell biological consequences of BTK signaling are varied and include survival, apoptosis, cytoskeleton, and transcriptional regulation. For example, related sequelae such as increased glucose uptake and protection from apoptosis result from BTK expression in breast cancer cells (Eifert et al., 2013). Another possible consequence of BTK signaling in cancer cells is the recently discovered involvement of BTK in activating the inflammasome through ASC phosphorylation (Weber, 2021). Inflammation is an underlying cause of several cancers (Karki et al., 2017), and BTK might modulate cancer progression in this way.

## Isoforms of Bruton’s Tyrosine Kinase as New Targets in Epithelial Tumors

Tyrosine kinases often execute key steps in pathways governing cellular proliferation, survival, differentiation, and motility (Blume-Jensen and Hunter, 2001; Giamas et al., 2010) and represent the largest chemotherapeutic target class. BTK inhibitors were originally developed for use in rheumatoid arthritis and other autoimmune diseases. In the past decade, these drugs have also been tested in clinical studies for efficacy in treating B-cell malignancies (Honigberg et al., 2010; Di Paolo et al., 2011; Byrd et al., 2013; Evans et al., 2013). Ibrutinib, an irreversible BTK inhibitor, was initially approved for the treatment of chronic lymphocytic leukemia (CLL) and mantle cell lymphoma (MCL) (Hendriks et al., 2014). Ibrutinib also has been tested to treat large B-cell lymphoma, follicular lymphoma, and multiple myeloma, among others. AstraZeneca and Acerta Pharma received Food and Drug Administration (FDA) approval in 2017 for the use of acalabrutinib in treating MCL. Additionally, BTK activity is suspected to play a central role in a number of other B-cell malignancies, including CLL, MCL, ALL, and multiple myeloma (Feldhahn et al., 2005; Hideshima et al., 2007; Mockridge et al., 2007; Cinar et al., 2013).

Bruton’s tyrosine kinase kinase activity has been shown to play pro-tumorigenic roles in a variety of epithelial tumors. By virtue of the fact that many of these are inhibitor studies, the specific isoform is not always known. In prostate cancer, BTK was found to be overexpressed in prostate cancer tissues and prostate cancer cells. The expression of BTK was correlated with prostate cancer grade in patient specimens and BTK promoted cell growth *in vitro* (Guo et al., 2014). In gastric cancer, BTK is over-expressed in gastric carcinoma tissues. Knockdown BTK or inhibition of BTK with ibrutinib blocked the growth of gastric cancer cells and increased cell apoptosis. Inhibiting BTK activation reversed gastric cancer cells’ resistance to docetaxel (Wang et al., 2016). In human ovarian cancer tissues, the expression levels of BTK correlated with the stages of disease. Higher BTK expression increased metastasis and was related to cisplatin resistance through regulation of ovarian cancer stem cells (Zucha et al., 2015). In glioblastoma multiforme (GBM), clinical glioma samples possess increased BTK expression when compared with normal brain cells. Inhibition of BTK activation with ibrutinib blocks proliferation, migration, and invasion ability of glioma cells in *in vitro* and *in vivo* modes (Wei et al., 2016; Wang et al., 2017). In colon cancer, p65BTK is overexpressed in colon cancer and interacts with the RAS/ERK pathway (Grassilli et al., 2016). All these findings provide evidence that isoforms of BTK are potential targets for the treatment of epithelium-derived tumors such as breast cancer, gastric carcinoma, ovarian cancer, GBM, and prostate cancer. Indeed, our studies show that several BTK inhibitors, including ibrutinib, AVL-292 (spebrutinib) and acalabrutinib, effectively inhibit breast cancer and prostate cancer cell growth (Kokabee et al., 2015; Wang et al., 2016). In most instances, the effective concentration of these drugs in inhibiting proliferation or survival of cells *in vitro* is tens of micromolar. Despite this, BTK inhibitors are currently being tested as single agents or in combination with other compounds in ongoing clinical trials focused on several epithelial tumor types.

Bruton’s tyrosine kinase inhibitors have impacts aside from decreased proliferation of epithelial cancer cells. Increased survival signaling and apoptosis suppression due to BTK expression has been shown to confer resistance to doxorubicin (Eifert et al., 2013) and lapatinib (Wang et al., 2016) *in vitro*. Activation of the PI3K downstream effector AKT pathway by NRG or EGF promotes lapatinib resistance in HER2^+^ breast cancer cells (Konecny et al., 2006; Weigelt et al., 2010; Wilson et al., 2012; Lovitt et al., 2015). BTK inhibition prevents lapatinib-resistant breast cancer clones from arising. In the case of ibrutinib, this may be in part due to its effect on HER2 kinase activity. Ibrutinib irreversibly binds to a cysteine residue (Cys-481) near the ATP binding pocket of BTK (Honigberg et al., 2010) and other kinases in the human genome including the EGFR family (Pan et al., 2007). Importantly, both NRG- and EGF-dependent growth factor-driven resistance to lapatinib is blocked by AVL-292 (spebrutinib), which has no effect on EGFR family activation. These results indicate that BTK signaling contributes to clinically significant ligand-dependent lapatinib resistance in HER2^+^ breast cancer. Since BTK is one of only two PH domains containing tyrosine kinases, its function in epithelial tumor cells may be to amplify critical survival signals downstream of PI3K signaling. However, the nature of these downstream signals is still poorly understood. One recently described possibility includes activation of DDX41 helicase activity by BTK phosphorylation in hematopoietic cells (Lee et al., 2015). DDX41 is universally expressed and inhibits translation of p21mRNAs by binding to the 3′ UTR (Peters et al., 2017). Since p21 localization and expression levels have been shown to impact drug resistance in cancer cells (Hawthorne et al., 2009; Koster et al., 2010), BTK activity could potentially work through this mechanism.

In addition to direct impacts on proliferation signaling, BTK activation triggers signaling events that alter cell intrinsic behaviors of B cells related to tissue homing and differentiation in the B-cell maturation process. It is possible that the BTK isoforms may function to regulate behaviors in solid tumor cells. For example, studies have shown that BTK signaling plays a critical role in the communication between osteoblasts and osteoclasts (Shinohara et al., 2008), and early steps in B-cell maturation occur in bone marrow microenvironments. BTK activity may impact how epithelial tumor cells interact with bone niches. Indeed, ibrutinib treatment significantly decreases metastatic dissemination of tail vein-injected breast cancer cells to the bone (Conklin et al., 2019); however, it is not known whether decreased osteotropism caused by BTK inhibition is a cancer cell autonomous effect. The inhibitor may, for example, affect bone remodeling by osteoclasts, which affects metastasis efficiency.

## Perspectives

Based on the evidence that BTK has multiple functions in B-cell malignancies including migration, survival, proliferation, and differentiation, we predict that BTK isoforms are likely to participate in a variety of processes related to the pathology of epithelial tumors. Going forward, the challenge will be to define their biological functions and identify relevant signaling pathways in epithelial tumors. Moreover, it will be interesting to investigate the effect of BTK inhibition on epithelial tumor biological behaviors *in vivo* including growth, metastasis, and differentiation such that current formulations of BTK could be used in the treatment of epithelial tumors.

## Author Contributions

XW wrote the manuscript. DC wrote and edited the manuscript. LK and MK contributed to the materials and information incorporated in the manuscript. All authors contributed to the article and approved the submitted version.

## Conflict of Interest

Some of the work reviewed has been used to support one or more patent applications owned by the Research Foundation for the State University of New York and is related to the current interests of Cancer Molecular Design Works, LLC in which DC and XW have financial interests. The remaining authors declare that the research was conducted in the absence of any commercial or financial relationships that could be construed as a potential conflict of interest.
